# On the Importance of Intraindividual Variability in Cognitive Development

**DOI:** 10.3390/jintelligence6020017

**Published:** 2018-03-22

**Authors:** Anik de Ribaupierre, Thierry Lecerf

**Affiliations:** Faculty of Psychology and Education Sciences, University of Geneva, 40 Boulevard du Pont d’Arve, CH-1205 Geneva, Switzerland; Thierry.Lecerf@unige.ch

Developmental cognitive psychology (as well as cognitive psychology in general) has a long-standing tradition to ignore all variations other than age, as if individual variations were only measurement error or noise. Even though Cattell [1,2] has drawn attention long ago to the necessity for psychology to consider at least three sources of variation in data collection: variables or situations, individuals, and occasions (the “Data Box”), the emphasis has essentially been placed on the influence of variables to the detriment of the individuals. In a very different perspective, Piaget focused on the “epistemic subject”, that is the theoretical core (rather than a mean subject, based on statistics) without any interest for individual differences [3]. A generalist focus was legitimate because of his epistemological interest for the development of knowledge rather than for the development of children [4,5]. This epistemological stance has not been retained by most theories suggested in developmental psychology in the last fifty years. Yet the focus has remained on general (usually statistical means), universal trends. Although between-individuals differences (interindividual variability) are often acknowledged, they have usually been relinquished to applied psychology if not considered as mere noise or measurement error. Moreover, little interest has been granted to within-individual variations (intraindividual variability) until recently. Psychology might actually be the last discipline to believe, at least implicitly, in some sort of universals as concerns the individuals. This is not the place to develop the role of diversity in other sciences, but let us just evoke physics with concepts such as complexity, dynamical systems or chaos, and catastrophe theory (for application to developmental psychology, see [6,7]), or biology with its interest for the variability of the living species (e.g., [8]).

The proposition to grant fundamental importance to individual variations in developmental and lifespan psychology is not new (see Wohlwill [9] for a precursor). Nesselroade [10] has warned against the danger of ignoring intraindividual variability (see also [11,12]). Molenaar [13,14,15] has very elegantly demonstrated, relying on simulations of factor analyses, that the ergodicity hypothesis does not hold for psychology. Phrased simply and applied to psychology, the hypothesis of ergodicity implies that the individual is similar to the group. However, Molenaar’s work shows that observations relative to interindividual differences (factor analyses) cannot be applied to an individual to draw the same conclusion about intraindividual variability. Note that this equivalence is an assumption implicitly held by most studies in psychology when the mean performance or the developmental trend of a group is considered to apply to all individuals composing that group. Recently, a renewed interest has emerged for variability, particularly in lifespan developmental psychology, and there are a number of empirical studies integrating individual variability (see examples below; see also [16]). Yet, the bulk of studies remains cross-sectional and centered on group means and univariate data (that is, data collected task-by-task). The present special issue aims at providing a broad picture (although of course not exhaustive) of how individual, in particular intraindividual, variability is presently addressed both theoretically and empirically, by gathering a number of authors who have contributed for quite some time to this increased interest in variability.

Several types of variability should be distinguished. Interindividual variability (also labeled diversity, see [17]) concerns differences between persons in a given group in at least one task; it should not be confused with age differences even though a number of experimental psychologists tend to classify age differences within interindividual differences [18]. Diversity is the type of individual variability that is most frequently acknowledged, although a focus on individuals remains rare; it generally suffices to report that individual differences are large or/and significant. Intraindividual variability (designated as IIV in the remainder of this text) concerns variations that occur within individuals, such as short-term fluctuations (labeled inconsistency) either within-task trial-to-trial variability, or in frequent repetitions of the same task within a relatively short period of time (measurement bursts), or across tasks (dispersion or heterogeneity), or across longer time periods (intraindividual change).

All papers in the present issue address one or/and the other type of intraindividual variability, while (a) presenting empirical data to demonstrate its importance; and (b) showing how IIV brings specific information relative to a centration on a central tendency. Together, the papers cover the entire lifespan, by presenting data on children (at least from school age upwards) and young and older adults. Most papers concern healthy individuals, and one also concerns non-healthy older adults.

In the first paper, Jacques Lautrey proposes a multiprocess framework; it opens the possibility to uncover and analyze the existence of different developmental pathways susceptible to account for phenomena that are usually considered as controversial results. He takes the example of the development of numerical cognition in young children as analyzed in a large number of empirical studies from the scientific literature in the field. Jacques Lautrey’s model is based on four fundamental concepts: (1) reconstruction by which primitive cognitive functions are transformed; (2) plurality, that is, the suggestion that many processes are simultaneously available to fulfill the same function, but not necessarily by using the same information; (3) interaction, notably between several of these processes; and (4) substitution, borrowed from Reuchlin’s [19] suggestion that various processes can function vicariously, that is, they would be available (unless damaged) in all individuals but might differ in their probability of activation depending on the individual and/or the situation.

The proposition that several processes co-exist within individuals as proposed by Lautrey is not new. However, the assumption of the simultaneous activation of several processes in a given task, and more importantly of their mutual interrelationships, is the signature of a multidimensional model, the term dimension being here understood as in factorial analyses. It can be contrasted with a unidimensional model, such as Piaget’s theory, but also of many other, more specific developmental theories, in which development in one task is considered to be subtended by a single process, identical for all individuals. The consequence of a unidimensional perspective is that children are all construed to develop along an identical path, and only differ by the speed of their development. In contrast, several developmental paths for different children may be envisaged within a multidimensional framework. Although most contemporary theories do no longer look for general mechanisms and hypothesize the existence of multiple, often very specific processes, it is still the case that they rarely address the question of whether development relies on different processes for different individuals. Moreover, the interrelationships between several processes are rarely considered. Interaction and substitution are conceived in Lautrey’s model as sources of within-individual variability and are therefore susceptible to account for interindividual differences in IIV. Also, all these processes and their interactions are inscribed within a dynamic systems perspective. Lautrey’s thorough analyses of studies dealing with the quantification of sets of discrete objects help in overcoming some contradictory findings in the abundant literature on that topic. Moreover, he also argues that another famous and controversial distinction in cognitive psychology, namely propositional or symbolic processing, on the one hand, and analogical processing, on the other hand, can yield both intra- and interindividual differences. A given task is not necessarily propositionally (or analogically) processed once and for all. Processing could be propositional for some children and analogical for others; it could also be analogical at one moment of development and propositional at another (see also [20,21,22]). Lautrey’s paper constitutes a very good introductory chapter to this special issue, because he defines concepts that can be applied to most empirical papers to follow, even though their respective authors do not necessarily or explicitly refer to the same processes as Lautrey.

In the second paper, Galeano-Weber, Dirk, and Schmiedek address IIV in (school age) children. They report part of an intensive microlongitudinal study included within a larger project; precision in a spatial working-memory task, with different memory loads, was assessed three times a day over a period of four weeks. Adopting such a complete and complex design allows for a number of novel theoretical and methodological contributions. First, the authors introduce a sophisticated procedure to study IIV in accuracy. Usually, inconsistency is measured in terms of Response Times (RTs) because RTs allow for computing an individual standard deviation across trials. Studies in which inconsistency in accuracy has been examined are rather rare because accuracy is often scored in binary terms (success/failure), which does not allow for devising a quantitative index of IIV. In contrast, in Galeano-Weber and collaborators’ paper, accuracy is assessed in terms of spatial recall precision (i.e., the distance between the participant’s reported and the true target location). Second, the authors refine the construct of IIV by distinguishing several temporal levels: (a) IIV across items (i.e., single responses within trials for the different elements to be stored in working memory); (b) IIV across trials, a trial consisting of two or three items, depending on the memory load; (c) IIV across occasions (three administrations by day); and (d) across days. Third, the authors used mixed models, which makes it possible to compare IIV at these different levels. As could be expected, considerable IIV was observed at all temporal scales. Yet, and very interestingly, only item-to-item variability correlated with a change in memory load. Moreover, this type of IIV was related to age (older children showing somewhat less variability), fluid intelligence, and school achievement. Such findings demonstrate the interest to further decompose IIV while also showing that using only an average performance is not sufficient (although of course simpler) to understand performance at the level of an individual.

Third, Perret and Dauvier also examine IIV in school age children by using response times in the Raven’s Progressive Matrices task; RTs are used here as a sort of proxy for strategies. It is indeed often not easy to study strategies, particularly with children, even though numerous studies have insisted on the importance (and necessity) of strategies in this well-known task. It has been shown in several studies (including the Perret and Dauvier one) that global RTs across the task (mean RT for instance) does not relate to performance, probably because it is too global. The authors’ hypothesis was that modulation of times as a function of item difficulty (i.e., responding more slowly in a difficult item) would be more appropriate. They devised an intraindividual index of modulation by computing individual correlations between an RT for an item and that item difficulty; hence, a high positive correlation for a given child reflects more time spent on a difficult item. Results indicated that the modulation index correlated not only with age but also with performance. RT modulation also functioned as a mediator for the relation between age and performance. Pushing further the analysis by using a generalized additive model, the authors found that the relation between RT and item difficulty was linear only for children presenting an efficient performance; RTs in less-efficient children increased between easy and intermediate items, but did not increase for the most difficult items, perhaps reflecting some kind of discouragement. Results were similar when a Rasch model was used to estimate the child’s ability instead of raw performance. The authors conclude that the modulation of study time is a key strategic factor for understanding developmental and individual differences. We wonder whether modulation is a strategy as such or whether it simply indicates that children use a more complex strategy. In any case, this study demonstrates very nicely (a) the need to go beyond a global index of time, such as the mean; and (b) that the profile of different children (efficient and less-efficient) differs qualitatively.

In the fourth paper, Hofman, Jansen, de Mooij, Stevenson, and van der Maas also present a study conducted with school age children. This study is truly striking by the size and length of the project within which it is inscribed. Inspired by an idiographic approach requiring a study at the level of the individual [14], the authors have developed several educational projects in which a novel method for monitoring and measuring computer adaptive testing (CAT) is used. These projects involved thousands of children in schools (or at home) on a daily or weekly basis. The authors have adopted a subtle measuring system making it possible to compromise between the number and difficulty of items in principle required by adaptive testing, on the one hand, and the discouragement that the task generated on the basis of these principles could generate in children, on the other hand. Interestingly, the scoring rule adopted is based on an adaptation of the Elo system used for the ranking of chess players, and combines speed and accuracy. The data presented in this paper come from a subset of addition and multiplication data obtained on children playing on a daily basis for 15 weeks, and the analysis focuses on intraindividual analyses (other analyses on interindividual differences have been presented in other papers). Results show a large variability across items despite their similarity in content. In a first set of analyses (learning analytics), the authors present the interest to (a) distinguish whether the child learns the item (switches between incorrect and correct responses); (b) describe the learning pattern; and (c) analyze the stability (and variability) of responses across time. We refrain here from reporting the number of players studied and the number of their responses! They are truly overwhelming. To summarize in a simplistic manner, the results indicate that irregularities in learning (e.g., switches to correct responses combined with frequent relapse to lower ability) were the rule rather than a smooth, improving learning pattern. In a second set of analyses, the authors address the question of the unidimensionality versus the multidimensionality of items, and show that additive items are not incompatible with a hypothesis of unidimensionality (all items addressing the same construct). In contrast, for multiplicative items, two types of items should be distinguished, which do not correlate with each other. Remember that all problems are relatively simple items played over 3 months on which basis one could think that they would be relatively similar. In sum, this paper demonstrates the usefulness of adopting an approach centered on the individual. Given its degree of methodological sophistication and the size of the data collected, it might lead to some feeling of helplessness in the reader (including us): Who can adopt such an approach in developmental studies? Probably few research groups, thus collaboration between different research groups might offer an alternative solution.

In the fifth paper, Joly-Burra, van der Linden, and Ghisletta deal with older adults. Their approach is novel in three aspects, in addition to stressing the importance of intraindividual variability. First, they assess prospective memory and inhibition within the same task, a Go/No Go task in two versions, focusing on intraindividual variability. Inhibition was indexed by the number of commission errors in the Go/No go task (incorrectly pressing the target key in a NoGo trial); prospective memory was measured by the number of omission errors. Second, they distinguish two types of IIV in RTs: amplitude of fluctuations measured by an intraindividual standard deviation (as is usually done in assessment of IIV), and time dependency based on time-series (asking whether RT at a given time is influenced by previous RTs). The latter type of IIV can be considered as assessing temporal dynamic effects. Third, they use a dynamic structural equations modeling to measure the joint effects of these two types of IIV (amplitude-based and time-dependent) together with the mean level. Results show that both higher latencies (mean RTs) and amplitude-based IIV were associated with inhibition failures, whereas time-dependent IIV predicted inhibition only at the beginning of the task. Then, inhibition was associated with prospective memory. There was, however, no association between either type of IIV and prospective memory performance. Of interest is the fact that the two types of IIV differed from one another in their correlational patterns; the authors suggest that the amplitude of fluctuations might be detrimental whereas time-dependent IIV could reflect the use of exploratory strategies to attain a better level; this second type of IIV would thus be functional.

The sixth article, by Halliday, Stawski, Cerino, DeCarlo, Grewal, and MacDonald, also presents data collected in older adults, and contains a clinical facet. They examine intraindividual variability across tasks rather than across trials; that is, they examine dispersion across a number of cognitive measures, comparing three groups: healthy older adults, an amnestic MCI group, and a small sample of carefully screened Alzheimer patients. A further objective was to relate dispersion with lifestyle activities (physical, social, and cognitive). The focus on dispersion is interesting, as this type of IIV has been much less investigated in older adults than inconsistency. Results showed that dispersion was already relatively large in healthy controls as other studies have also shown [23]. Yet, dispersion was significantly larger in Alzheimer patients than in both healthy controls and MCI patients. Using discriminant analyses, the authors also observed that dispersion was a significant predictor in examining the risk of being classified as Alzheimer (but not the risk of MCI) relative to healthy controls. A more engaged lifestyle was associated with a reduced likelihood of being classified as Alzheimer or MCI. When studying the joint effect of dispersion and of lifestyle, the authors noted that dispersion remained predictive of Alzheimer, whereas lifestyle remained predictive of MCI. As the authors note, an analysis in which the lifestyle activities would be further decomposed would be interesting. Furthermore, it would be crucial to assess whether inconsistency and dispersion correlate or are independent from one another; such a comparison does not seem possible here: Most tasks (currently used in neuropsychological assessments) provide only global scores and most probably do not contain enough trials to compute trial-by-trial variability. The larger project within which the present study was included might hopefully contain a few tasks in which inconsistency can be computed and then compared with dispersion.

The seventh paper, by Fagot, Mella, Borella, Ghisletta, Lecerf, and de Ribaupierre, reports abundant data documenting age differences in inconsistency (across trials IIV) over the lifespan (primary school age children to older adults) in several tasks. It is important to note that the tasks were identical for all participants, making it possible to compare age trends. A further objective was to contrast inconsistency measures in latencies in processing speed tasks, on the one hand, and in accuracies in working memory (WM) tasks, on the other hand. There are indeed some controversial results in the literature: A number of authors did not observe age differences in inconsistency in accuracy scores. Computing inconsistency in relatively complex tasks in which accuracy scores are used is often not possible because accuracy is usually scored in binary terms (success/failure), as also noted in the Galeano Weber et al.’s paper. An intraindividual standard deviation cannot then be computed unless using response times again, or simply percentage of success across blocks of trials; the latter solution is in turn not informative in an adaptive task because it only indicates whether the task is adapted to the participant’s level of performance. In the present study, WM tasks were adaptive; this made it possible to administer a large enough number of trials of the same complexity to compute a standard deviation on the number of correct responses across trials whether the response was fully correct or not. Beyond the existence of a large IIV in all tasks (this should not come as a surprise by now for the reader), clear differences between age groups were observed. For all RT tasks, children were the most variable, then the older adults, and the least variable were young adults. There were a few further age differences, depending on the task: younger children (9–10 year-olds) were more inconsistent than older ones (11–12 year-olds), and young-old adults (between 60 and 70 years of age) showed less inconsistency on some tasks than older-old adults (over 70 years of age). In contrast, in the WM tasks, the differences between age groups were not significant in most comparisons; moreover, the descriptive statistics showed a tendency for the young adults to be more inconsistent. This difference between the two types of tasks could of course be linked to the type of scores: A high value in an RT task is associated with being slower (poorer performance), whereas a high value in the WM tasks is associated with a better performance. As a result, higher IIV might directly reflect the mean level. Yet, intraindividual standard deviations were all computed on values residualized for the participant’s mean; therefore, they should be relatively independent from the performance level. There seems to be a more profound difference between the two types of tasks, leading the authors to suggest that inconsistency might be dysfunctional in the RT tasks as is often argued, but functional or adaptive in the WM tasks because it would index changes in strategies.

Finally, the paper by Mella, Fagot, Renaud, Kliegel, and de Ribaupierre is issued from the same project as the previous one, but reports on a longitudinal facet that was conducted on the older adults only over a period of approximately 7–8 years. It centers on the individual patterns of change observed in the first and the last (fourth) wave of assessment. The objective of the authors was to focus on the individual using an idiographic approach. Some longitudinal studies mention the existence of (significant) interindividual differences in trajectories, but almost no study defines change at the level of the individual. The reason why there are so few studies focusing on intraindividual change is probably because the reliability of such change is not assured. For instance, in an RT task, it is of course insufficient to observe a 20-millisecond difference (or any other higher value) in the mean response over several years for considering that there is a significant change. A frequent solution consists in relying on a standard error of measurement (SEM); yet SEM is defined at the group level and not at the individual level. As a result, a single individual might be considered to have changed when included in a given sample, but not if he was included in another group. The authors propose two novel methods to assess change within individuals, both made possible because a relatively large number of trials was used in all tasks: estimate a bootstrap-based confidence interval and individual analyses of variance. The former method was used to determine for each pair of assessments—the paper reports on waves 1 and 4—whether the individual significantly declined, improved, or remained stable. The latter method made it possible to assess the degree of heterogeneity of change across the tasks. Only the RT tasks of the project could be used, because the WM tasks did not contain enough identical trials (10 by condition and complexity level) to obtain estimates in the bootstrap-based approach. Results showed, task-by-task, that all three patterns of change (stability, increase, decrease) were obtained in almost all individuals. This demonstrates clearly that any longitudinal group curve does not reflect the participants that compose it and illustrates Molenaar’s claim that a hypothesis of ergodicity cannot be adopted in developmental psychology. Trajectories differed widely among individuals. Decline was more frequent when considering all the tasks and over 8 years but still far from being the rule. For instance, there were only 3 individuals out of 92 who showed decline in the nine conditions analyzed. Moreover, the analyses of variance demonstrated a large heterogeneity of change, meaning that for a given individual, change may differ (quantitatively or qualitatively) considerably from task to task. The data offer an empirical demonstration of the necessity to focus on the individual, and a strong support for Nesselroade’s [10,24] repeated claim that intraindividual variability should be examined seriously, and for Molenaar’s manifesto on the necessity for psychology to adopt an idiographic approach [14,25].

In sum, the present special issue offers a wide array of approaches to the study of intraindividual variability. Not only does it present trial-to-trial fluctuations (inconsistency), the type of IIV most commonly reported in the literature (although certainly not yet sufficiently represented), across-tasks variability (dispersion), and across-years variability (longitudinal, intraindividual change), but it also offers novel openings to IIV, such as time-dependency (Joly-Burra et al.’s paper) and variability at different time scales (Galeano-Weber et al.’s paper). Together, the papers demonstrate that variability is observed at all age periods of the life span. We consider that the present papers represent very well this domain of research in full expansion—or so do we hope—and want to thank all our colleagues to have played the game.

All the researchers working on IIV have one day or another encountered some doubt or even opposition as to the novelty or usefulness of such an approach. The field of developmental psychology still consists in its majority of cross-sectional studies, using often a single task with a few small groups, and of statistical analyses centered on group analyses. Anecdotic but very illustrative, we read recently the following statement in a review: “*The findings make a compelling case that intraindividual variability exists but not such a strong case that it matters. Said another way, measuring such variability often increases the testing burden on participants and researchers alike, sometimes substantially. What deep theoretical insights are likely to justify the extra effort? Many readers may conclude something like, ‘Yeah, interesting, but not worth the time, effort, and cost*”. We hope that, together, all the papers presented in this special issue will convince our readers not only to consider IIV as an existing reality and to contribute to its study, but also that it matters theoretically. They show that IIV contributes other information relative to the mean, sometimes complementary, sometimes very different. Perhaps, the sophisticated statistical models and/or the abundance of data in certain studies may induce some discouragement in the reader: “*my group and I will just not be able to conduct such research*”. If a better understanding of the meaning of such variability does indeed require large data sets and new methods, the field is still in need of more data to offer some counterpart to the decades of research spent in restricting research on means and (small) groups. Also, it is time that groups of labs be formed and collectively contribute to this novel way of collecting data.
